# Effects of Type I Collagen Degradation on the Durability of Three Adhesive Systems in the Early Phase of Dentin Bonding

**DOI:** 10.1371/journal.pone.0116790

**Published:** 2015-02-17

**Authors:** Lin Hu, Yu-hong Xiao, Ming Fang, Yu Gao, Li Huang, An-qi Jia, Ji-hua Chen

**Affiliations:** 1 State Key Laboratory of Military Stomatology, Department of Prosthodontics, School of Stomatology, Fourth Military Medical University, Xi’an, 710032, China; 2 Department of Stomatology, Kunming General Hospital of Chengdu Military Command, Kunming, 650032, China; Nanjing Medical University, CHINA

## Abstract

**Objective:**

This study was designed to evaluate the effects of type I collagen degradation on the durability of three adhesive systems in the early phase of dentin bonding.

**Methods:**

Bonded dentin specimens were prepared using three different types of adhesive systems. Micro-tensile bond strength and degradation of collagen were tested before, and after 1 month or 4 months of aging in artificial saliva. The relationship between micro-tensile bond strength and collagen degradation was analyzed by calculating their Pearson’s correlation coefficient.

**Results:**

Aging induced time-dependent reduction in micro-tensile bond strengths for all the tested adhesive systems, although such reduction for the single-step self-etching adhesive G-Bond (GB) was not statistically significant. The bond strength of the two-step self-etching primer adhesive system Clearfil SE Bond (SEB) was similar to that of the two-step etch-and-rinse self-priming adhesive system Single Bond 2 (SB), and they were both significantly reduced after one or four months of aging. A negative correlation was found between the degree of collagen degradation and magnitude of micro-tensile bond strength (*r* = - 0.65, *p* = 0.003). The Pearson’s correlation coefficient was 0.426, indicating that 42.6% of the aging-induced reduction in bond strength can be explained by the degradation of collagen.

**Conclusions:**

In the early phase of dentin bonding, there was a negative correlation between the degree of collagen degradation and the magnitude of micro-tensile bond strength. The reduction of bond strength was accompanied by the degradation of collagen. These results provide evidence for the causative relationship between the degradation of collagen and the deterioration of dentin-adhesive interface.

## Introduction

The bonding between adhesives and dentin can be regarded as a process of materials exchange [1]. The minerals of dentin are replaced by resin monomers, which permeate into the porous collagen scaffold exposed by demineralization and subsequently polymerize to envelope the collagen fibrils and form inter-locking microstructures that provide bonding strength. Such a structure composed of resin-matrix reinforced collagen fibrils is termed as hybrid layer [2].

Compared with enamel, dentin contains more water and organic components, especially type I collagen. Such characteristic of dentin makes it difficult to be infiltrated by hydrophobic monomers, and thus renders the successful bonding of dentin challenging [3]. Type I collagen accounts for 30% (volume) of the mineralized dentin tissue and 90% of the dentin organic matrix. The major collagen fibrils of type I collagen with the diameter of 70 nm to 90 nm are connect with the minor branching fibrils of noncollagenous proteins which has diameters of 20 nm to 40 nm, forming a typical collagen banding of 67 nm [4].

Since adhesion is resulted from the infiltration of resin monomers into the dentin substrate, the stable and durable bonding can be achieved if the exposed collagen network is fully encapsulated by the adhesive resin to protect against degradation. Thus, collagen fibrils and polymerized resin that encapsulate the collagen fibrils are recognized as two main components of the bonding interface. Age-related biodegradation of dentin collagen matrix and hydrolysis of adhesive resin within the hybrid layer are the major reasons for the deterioration of dentin-adhesive interface [5–7]. However, the extent of influence of type I collagen degradation on durability of dentin bonding is not certain.

Several studies have provided morphological evidences for the hydrolysis/zymohydrolysis of collagen matrix after long-term storage [8–11]. One group of researchers evaluated the durability of resin-dentin bonds after aging in water for as long as 10 years and found remarkable reduction of bond strength after aging [12]. However, research about the influence of collagen degradation on durability of bond strength in the early phase of dentin bonding is scarce. The statistical relationship between the collagen degradation and dentin-adhesive interface has not been established yet.

Therefore, the purpose of this study was to provide experimental evidence for the negative effects of collagen degradation on dentin bonding and establish the specific correlation ship. In the present study, we monitored the extent of degradation of type I collagen by measuring the amount of hydroxyproline using an enzyme linked immunosorbent assay (ELISA), and tested the microtensile bond strength of several adhesives to dentin after various periods of aging.

## Methods and Materials

### 1. Ethics Statement

The study was approved by the Ethics Committee of Fourth Military Medical University. Informed consent (written) was obtained for use of tissue samples from the patients in this study.

### 2. Specimen preparation

For this study, sixty human permanent third molars (extracted within 1 month) were collected and stored in saline at 4°C after removing of soft tissues.

The occlusal enamel were removed by using a low-speed diamond saw (EQ-SYJ-150，MTI Co., USA) under water cooling. The exposed dentin surface was ground with 600-grit abrasive paper for 60 s under wet conditions to produce a standard smear layer [13], and the prepared specimens were stored in PBS (phosphate buffered saline, 1 mol/L disodium phosphate anhydrous 720 ml and 1 mol/L sodium dihydrogen phosphate 280 ml, pH 7.2) at 4°C.

Three different adhesive systems, namely the one-step self-etching adhesive G-Bond (GB, GC, Japan), the two-step self-etching adhesive system Clearfil SE Bond (SEB, Kuraray, Japan) and the two-step etch-and-rinse self-priming adhesive Single Bond 2 (SB, 3M ESPE, USA), were tested in this study. The basic compositions and application procedures of these adhesives are listed in Table 1 and Table 2, respectively.

**Table 1:** Composition of the adhesive systems used in this study.

Adhesive (Manufacturer)	Chemical formulations
Self-etching primer systems	
G-Bond (GC, Tokyo, Japan)	4-MET, UDMA, water, acetone, filler, photoinitiator
Clearfil SE Bond (Kuraray, Osaka, Japan)	Primer: MDP, HEMA, water Bonding resin: Bis-GMA, HEMA, 10-MDP, water, microfiller, camphorquinone
Total-etching adhesive systems (Etch-and-Rinse systems)	
Single Bond 2 (3M/ESPE, St. Paul, MN, USA)	Bis-GMA, HEMA, water, ethanol, photoinitiator

4-MET: 4-methacryloxyethyl trimellitate

UDMA: Urethane dimethacrylate

Bis-GMA: Bis-phenol A diglycidylmethacrylate

HEMA: 2-hydroxyethyl methacrylate

10-MDP: 10-methacryloxydecyl dihydrogen phosphate

**Table 2:** Application procedures of the adhesive systems.

Adhesive system	Application procedures
G-Bond	Apply adhesive with small sponge for 10 s, leave for 10 s, air to homogeneous surface for 5 s at the distance of 20 cm, light cure for 10s.
Clearfil SE Bond	Apply primer with small sponge for 20 s, leave for 20 s, air dry for 10 s, apply adhesive for 10 s, leave for 10 s, air to homogeneous surface for 10 s at the distance of 20 cm, light cure for 10s.
Single Bond 2	Etch for 15 s with 37% phosphoric acid gel, rinse for 30 s, air dry for 30 s, rewet bonding surface with 5 μl deionized water, apply adhesive with small sponge for 10 s, leave for 10 s, air thin to homogeneous surface for 10 s at the distance of 20 cm, light cure for 10 s.

The prepared specimens were randomly assigned to the three adhesive groups and the control group. The adhesives were applied on the dentin surface according to the manufacture’s instructions and resin composite (Lot: N145596, Filtek Z250 A3, 3M ESPE, USA) build-ups with the height of 2 mm were constructed layer by layer. Each layer of composite was light cured for 40 s with a Spectrum 800 light-curing unit which was previously tested (Dentsply, USA).

Fifteen specimens within each group were then randomly divided into three subgroups. Teeth in one group were tested for micro-tensile strength and collagen degradation immediately after 24 hours of storage in deionized water at 4°C. The other ten teeth, five in each group, were aged in artificial saliva for either 1 or 4 months at 37°C before subjected to micromechanical and collagen degradation assay. The main components of artificial saliva solution are shown in Table 3.

**Table 3:** Composition of the artificial saliva solution.

Composition	Content
Carboxymethylcellulose sodium	10 g
Sorbitol	30 g
Potassium chloride	1.2 g
Magnesium chloride	0.052 g
Sodium chloride	0.9 g
0.053% Calcium phosphate	200 ml
0.2% Sodium phosphate	10 ml
Paraben	0.33 g
Distilled water	1000 ml

### 3. Micro-tensile bond strength measurement

By using a slow-speed diamond saw, the bonded specimens were serially sectioned parallel to the long axis of the tooth under water cooling. Twenty beams with the cross-sectional area of 1 mm^2^ were harvested from each group. The height of dentin and resin composite in each sectioned beam was approximately 2 mm. The beams were loaded to a universal testing machine (AGS-500, Shimadzu Co., Japan), and the micro-tensile bond strength was tested at a cross-head speed of 1.0 mm/min [14].

### 4. ELISA for collagen degradation determination

After micro-tensile bond strength test, the failed samples were checked under sterecmicroscope (Nikon SMZ1500, Nikon Instruments, Japan) and specimens with “adhesive failure” were collected in each group. Then, the selected samples were frozen in liquid nitrogen and then milled into powder at -120°C [15]. The powder was collected, sieved through a 20 μm mesh and then kept in a desiccate until used. An aliquot of 10 mg dentin powder from each group was soaked in 1.0 ml of deionized water for 24 h at 37°C with continuous gentle agitation to prepare collagen extract. The extract was centrifuged for 15 min at 4°C (10000 r/min) and the supernatant was harvested for collagen degradation test. The amount of degraded collagen was measured by means of the rat anti-human Hydroxyproline ELISA kit (E0621h, Uscnlife, USA) [16, 17].

### 5. Correlation analysis between micro-tensile bond strength and collagen degradation

Two beams were randomly selected within each experimental group. After micro-tensile bond strength test, the same sample was used for collagen degradation measurement. Then, data were used for correlation analysis.

### 6. Morphological observations for hybrid layer and collagen with immunohistochemistry

The beams for morphological observations were randomly selected within each experimental group. After blocked in normal goat serum (ab7481, Abcam, UK) in PBS for 30 minutes at room temperature, the beams were incubated with a monoclonal anti-type I collagen antibody (C2456, Sigma, USA) overnight at 4°C. Washed in PBS, gold labeling was performed with a goat IgG anti-mouse IgG conjugated with 20-nm-diameter colloidal gold (ab27242, Abcam, UK) for 90 minutes at room temperature. After fixed, dehydrated and critical point-dried, the beams were subjected to coating with a 5 nm thick layer of evaporated carbon (JEE-420, JOEL, Japan). Observations were performed under a field emission in-lens SEM (JSM-6700F, JOEL, Japan) at 5.0 kV acceleration voltage. Final images were obtained by mixing both secondary electron and back-scattered signals.

### 7. Statistical analysis

After test for homogeneity of variance and normal distribution, the data was subjected to two-way (adhesives vs. time) analysis of variance (ANOVA), using SPSS 16.0. LSD-*t* test was performed for multiple comparisons. Pearson’s correlation coefficient was calculated and used for explaining the relationship between micro-tensile bond strength and collagen degradation values. Statistical significance was defined as *p* < 0.05.

## Results

### 1. Micro-tensile bond strength test

The mean bond strengths of the three tested adhesive systems before and after aging are shown in Table 4. The immediate bond strength of GB was approximately one sixth lower than that of SEB and SB (*p*<0.05).

**Table 4:** Bond strength of the tested materials (n = 20, x̄ ± s).

Adhesive system	Bond strength (MPa)		
	Immediate	One-month storage	Four-month storage
GB	24.07 ± 7.47 ^a^	22.11 ± 7.16 ^a^	20.55 ± 6.06 ^a^
SEB	29.53 ± 7.13 ^b^	28.88 ± 10.52 ^b^	18.96 ± 5.31^a^
SB	29.81 ± 7.34 ^b^	28.25 ± 9.32 ^b^	21.21 ± 6.39 ^a^

^a-b^ The same superscript letter represents no statistical difference.

The bond strengths of all three adhesive systems were reduced in a time-dependent manner after aging, although the reduction for specimens bonded with GB were not statistically significant. After one month of aging in artificial saliva, teeth bonded with GB exhibited micro-tensile bond strength of 22.11±7.16 MPa, which was significantly lower than that of SEB and SB (*p*<0.05). After four months of aging, the bond strength for SEB and SB were reduced to approximately two third of the immediate value, and they presented no significant difference as compared to that of aged GB. SEB and SB showed similar bond strength at every tested time point.

The results of two-way (adhesives vs. time) analysis of variance (ANOVA) are shown in Table 5. Both the type of adhesive system and the period of aging were found to have significant influence on micro-tensile bond strength (*p* < 0.05). No interaction between these two factors was observed.

**Table 5:** Results of two-way (adhesives vs. time) analysis of variance (ANOVA) (α = 0.05).

Source	df	Mean Square	F	Sig.
Adhesive system	2	304.913	5.328	0.006
Aging time	2	972.432	16.993	0.000
Adhesive system*Aging time	4	105.778	1.848	0.122

### 2. Collagen degradation assay

The results for collagen degradation before and after aging are presented in Table 6. Specimens in the control group, which was not bonded, exhibited very low level of collagen degradation and the amount of degraded collagen was similar at all three tested points. The immediate collagen degradation for specimens bonded with GB was 0.18 ± 0.01 μg/mg, which is significantly higher than that for SEB and SB. After one month of aging, collagen degradation was significantly increased in the SB group (*p* < 0.05). Extending the aging period to four months further enhanced the degradation of collagens in all adhesive groups, and all the values presented significant difference as compared to immediate results (*p* < 0.05).

**Table 6:** Collagen degradation of each group measured by ELISA (n = 6, x̄ ± s).

Adhesive system	Collagen degradation (μg/mg)		
	Immediate	One-month storage	Four-month storage
GB	0.18 ± 0.01^a^	0.19 ± 0.02^a^	0.26 ± 0.01^b^
SEB	0.16 ± 0.01^c^	0.16 ± 0.01^c^	0.21 ± 0.02^d^
SB	0.15 ± 0.01^c^	0.18 ± 0.01^a^	0.19 ± 0.02^a^
Control	0.05 ± 0.01^e^	0.05 ± 0.01^e^	0.06 ± 0.01^e^

^a-e^ The same superscript letter represents no statistical difference.

The results of two-way (adhesives vs. time) analysis of variance (ANOVA) are shown in Table 7. Both the type of adhesive system and the period of aging were found to have significant influence on the amount of collagen degradation of bonded dentin specimens (*p* < 0.05).

**Table 7:** Results of two-way (adhesives vs. time) analysis of variance (ANOVA) (α = 0.05).

Source	df	Mean Square	F	Sig.
Adhesive system	3	0.083	5.328	0.000
Aging time	2	972.432	0.014	0.000
Adhesive system*Aging time	6	105.778	0.002	0.000

### 3 Correlation analysis

**Fig 1:**
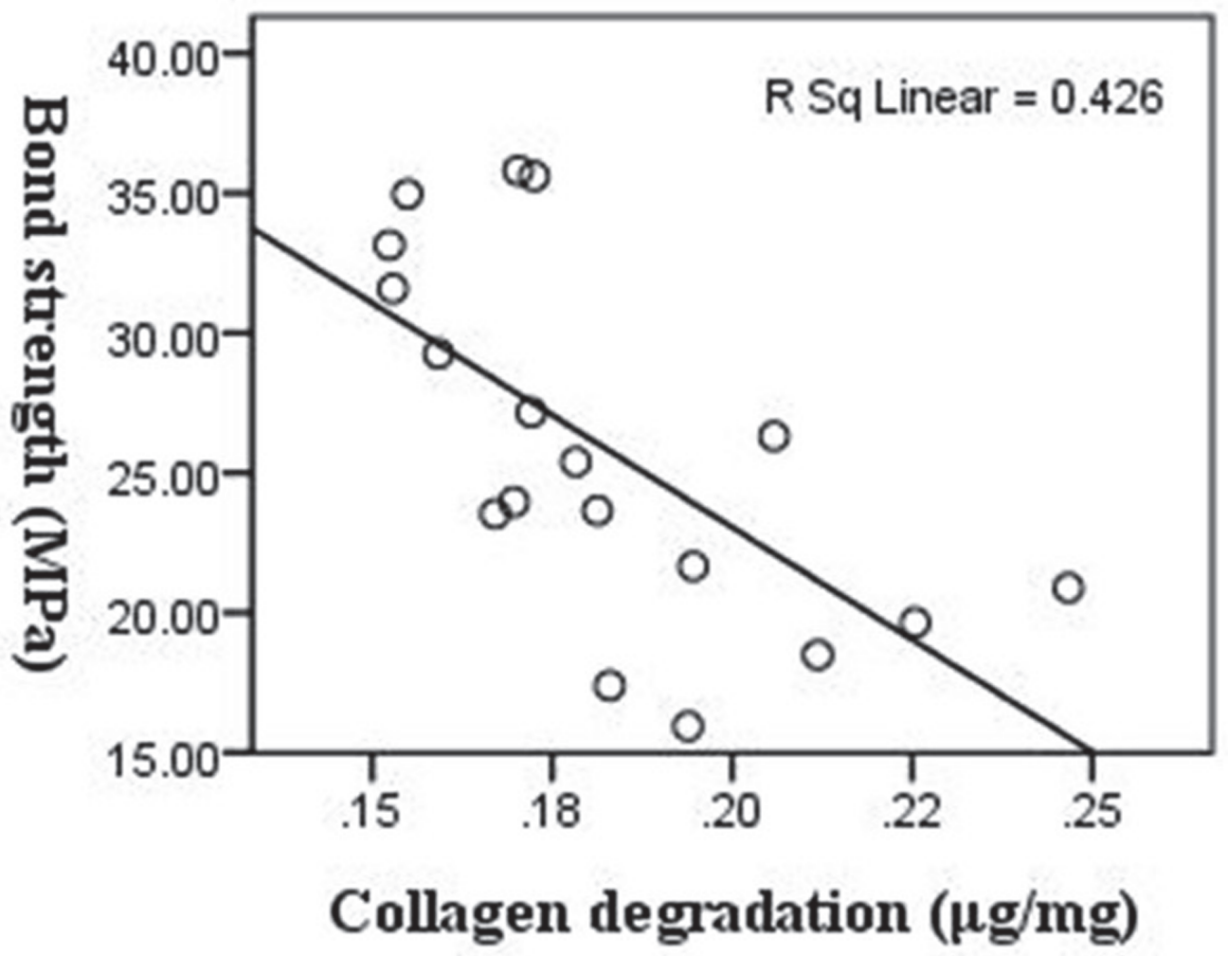
Scattered plots of linear analysis regression showing negative correlation between collagen degradation and μ TBS.

Negative correlation between the degradation of collagen fibrils and the bond strength of dentin restorations is shown in Fig. 1 (*r* = －0.65, *p* = 0.003).

### 4. Typical FEISEM images of the hybrid layer and collagen

**Fig 2:**
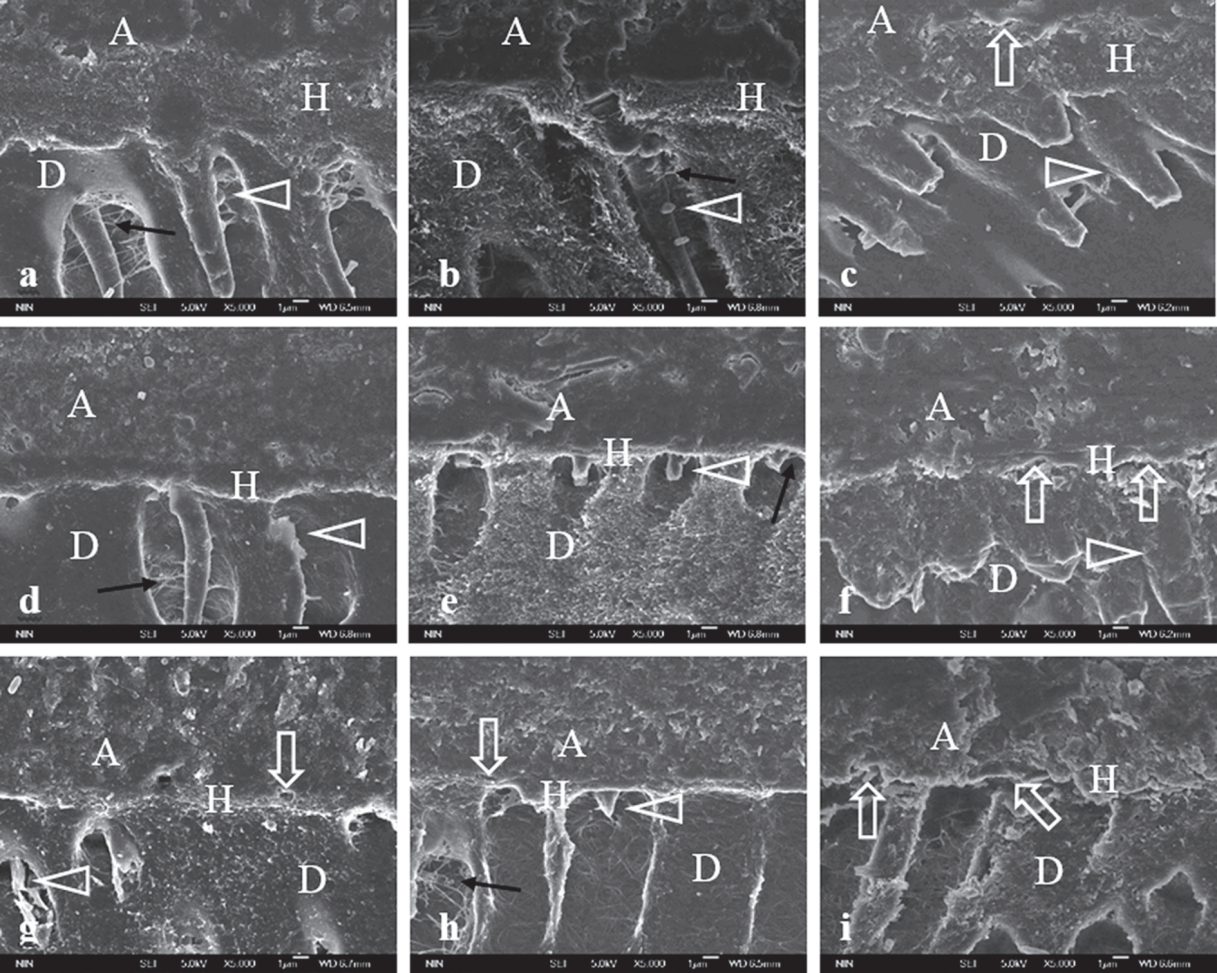
FEISEM micrographs of the hybrid layer. (×5 000). A = adhesive; H = hybrid layer; D = underlying mineralized dentin. a, b, c stand for dentin-SB interface without aging, aged for 1 m and aged for 4 m respectively. d, e, f stand for dentin-SEB interface without aging, aged for 1 m and aged for 4 m respectively. g, h, i stand for dentin-SEB interface without aging, aged for 1 m and aged for 4 m respectively. The non-uniform thick hybrid layer with long resin tags (open arrowhead) are clearly seen. There are lots of resin tags pointing to the lateral wall of the tubule with collagen fibril emanating from the resin surface (black arrow) (Fig. 2A). No visible gaps can be detected. There are fewer and shorter resin tags (open arrowhead) which interact with collagen fibrils pointing to the lateral wall of the tubule (black arrow) (Fig. 2B). Slight cracks (arrow) can be seen within the hybrid layer. Despite of large quantities of resin tags (open arrowhead) infiltrating into dentin tubules, no lateral branch of resin tags can be found (Fig. 2C). The hybrid layer is compact with numerous long resin tags permeate into the dentin tubules. There are fewer lateral branch of resin tags (open arrowhead). However, the main part of resin tags (black arrow) interacting with collagen is evident (Fig. 2D). Compact and homogenous hybrid layer with a great deal of resin tags (open arrowhead) are clearly detected. Resin tags are winded around by collagen fibrils (black arrow) (Fig. 2E). The structure of hybrid layer is non-uniform, while a great number of resin tags (open arrowhead) permeated into the dentin matrix. Cracks (arrows) along hybrid layer can be clearly detected (Fig. 2F). There are a few porous defects (arrow) within the certain part of hybrid layer. Resin tags, some of which are fractured (open arrowhead), can be clearly observed in the dentin tubules (Fig. 2G). Some porous defect (arrow) can be observed. There are short resin tags infiltrating into the dentin tubules (open arrowhead), collagen fibril under the hybrid layer (black arrow) (Fig. 2H). There is no clear boundary of the hybrid layer and the whole interface exhibited an inhomogeneous morphology with evident cracks (arrows). The dentin tubules were empty and no resin tags can be found (Fig. 2I).

**Fig 3:**
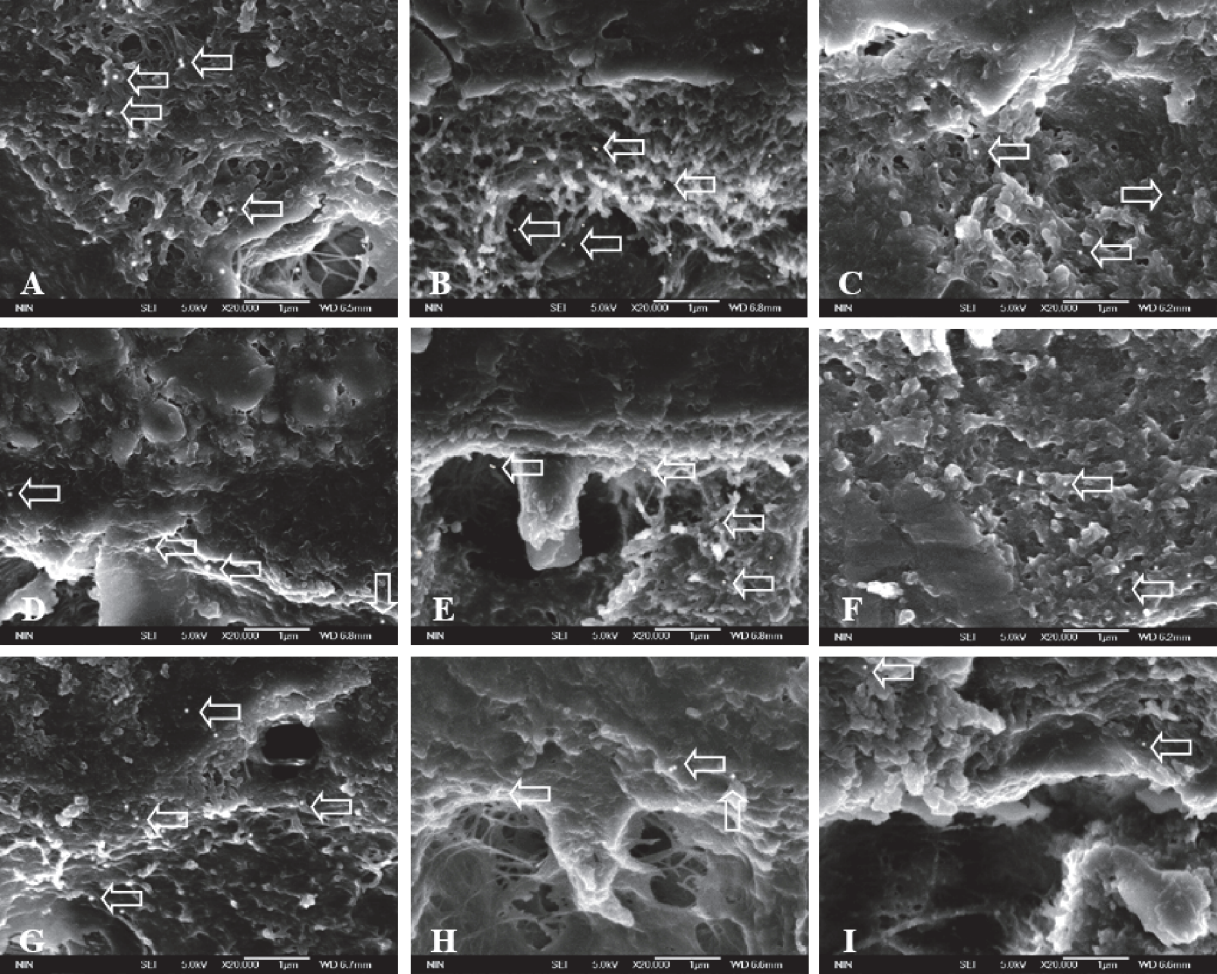
FEISEM micrograph of collagen with immunohistochemistry. (×20 000). A, B, C stand for dentin-SB interface without aging, aged for 1 m and aged for 4 m respectively. D, E, F stand for dentin-SEB interface without aging, aged for 1 m and aged for 4 m respectively. G, H, I stand for dentin-SEB interface without aging, aged for 1 m and aged for 4 m respectively. Arrows stand for collagen fibrils labeling. There are intense collagen fibrils labeling throughout the hybrid layer (Fig. 3A). Fewer labeling can be found throughout the hybrid layer (Fig. 3B). The labeling decreases significantly (Fig. 3C). Fewer positive labeling with uniform distribution are observed (Fig. 3D). Weak labeling can be observed (Fig. 3E). There are weaker gold labeling (Fig. 3F). There are intense uniform distribution of colloidal gold particles through hybrid layer (Fig. 3G). There are the resin tags permeating into the collagen fibrils network with a small quantity of labeling localized around it (Fig. 3H). Scarce labeling is present (Fig. 3I).

The images of the hybrid layer and collagen created with the three adhesives are shown in Figs. 2 and 3.

The SB adhesive applied onto dentin was characterized by the non-uniform thick hybrid layer with long resin tags and collagen fibril emanating from the resin surface (Fig. 2A). Intense collagen fibrils labeling throughout the hybrid layer could be observed (Fig. 3A). After aging for one month, no visible gaps and fewer labeling could be detected (Fig. 2B and 3B). After aging for four months, slight cracks can be seen within the hybrid layer and no lateral branch of resin tags could be found (Fig. 2C). The labeling decreases significantly (Fig. 3C).

The dentin-SEB interface represented compact hybrid layer and fewer lateral branch of resin tags (Fig. 2D and E). However, fewer positive labeling could be observed (Fig. 3D and E). Cracks along hybrid layer could be clearly observed after four months aging. However, the resin tags permeating into the dentin matrix with weaker gold labeling were obvious (Figs. 2F and 3F).

As can be seen from Fig. 2G, resin tags were formed in dentin tubules and some were fractured. Intense uniform distribution of colloidal gold particles could be found (Figs. 2G and 3G). After aging for one month, some porous defect could be occasionally observed and the numbers of resin tags that infiltrate the dental tubules with a small quantity of labeling were reduced (Figs. 2H and 3H). In the image of the bonded interface after four months of aging, no clear boundary of the hybrid layer could be detected, and the interface revealed an inhomogeneous morphology with evident cracks. No resin tags could be observed in any dentin tubules and scarce labeling was present (Figs. 2I and 3I).

## Discussion

The major finding of this study was that in the early phase of dentin bonding, there was a negative correlation between the degree of collagen degradation and the magnitude of micro-tensile bond strength. The reduction of bond strength over time is accompanied by the degradation of collagen fibrils, and 42.6% of the reduction in bond strength can be explained be the degradation of collagen (coefficient of determination R^2^ = 0.426).

While efficient immediate bond of various adhesive systems to dentin has been confirmed by numerous researches, the long-term durability of the dentin restorations is still questionable. Thus, there is a definitely need to test bonding effectiveness upon aging of the specimens [18]. In many researches about the aging of bonded interface, the tooth specimens were first sectioned into beam-shaped micro-specimens before immersing them into various aging solutions with the intention to accelerate aging effect [19]. However, such an aging protocol is not very closely related to clinical situations, and a recent study demonstrated that aging of the whole bond tooth, rather than sectioned beams, can better reflect and imitate the clinical performances of the restoration [20]. Therefore, in this study, the bonded teeth were aged in artificial saliva without sectioning.

The results of this study indicated that both the types of adhesive system and the period of aging have significant influence on collagen degradation. All three tested adhesive induced significant collagen degradation after four months of aging, and, among them, GB exhibited the highest amount of collagen degradation, indicating that the GB bonded specimens have relatively poor long-term stability.

In this study, tooth specimens bonded with SB experienced significant degradation of collagen in the first month of aging. Extending the aging period to four month only slightly further enhanced collagen degradation. Such phenomenon may be explained partially by the usage of 37% phosphoric acid gel. Presumably, etching prior to adhesive application may activate the intrinsic matrix metalloproteinases (MMPs) in dentin matrix, and subsequently accelerate the degradation of collagen [21, 22]. Furthermore, it has been demonstrated by Scott *et al* that the water in the interfibrillar spaces and the proteoglycans in acid-etched dentin can form hydrogels [23], which may work as a “molecular sieving” restricting the infiltration of adhesive monomers with relatively large size (such as Bis-GMA) [24, 25]. Since only small hydrophilic monomers like HEMA can penetrate through the depth of collagen network and there is a discrepancy between the depth between demineralization and monomer infiltration, the deep layer of the hybrid layer is very susceptible to water adsorption and subsequent hydrolysis. Collagen fibrils that are not encapsulated by polymer, as well as those exposed after hydrolysis of the hydrophilic monomers, can be subsequently degraded by intrinsic MMPs and eventually results in the failure of the bond.

Collagen degradation in the first month for tooth specimens bonded with GB or SEB was not so remarkable as compared to that of SB. This may be related to their relatively lower acidity compared with 37% phosphoric acid. Although there is no discrepancy between the depth of demineralization and monomer infiltration for self-etching adhesive which demineralize and infiltrate dentin simultaneously, the hydrophilic nature of these adhesives rendered them very susceptible to water adsorption, nanoleakage and monomer degradation [26]. The degradation of the monomers encapsulating collagen fibrils will eventually result in secondary exposure and degradation of collagen fibrils. The one-step self-etching adhesive system GB, which contains large amount of water, exhibited higher amount of collagen degradation than that of SEB, possible due to phase separation in the bonded interface [27]. Although the function monomer 4-MET in GB can form chemical bond with hydroxyapitite, this chemical bond is less insufficient and thus less stable in water as compared to that formed by 10-MDP [2], a patented function monomer in SEB. Since the chemical bond provided by 10-MDP is stronger than that of 4-MET, SEB can better protect the collagen fibrils from degradation than GB.

Phase-separation or the formation of micro-size bubble in GB may be the primary reason for its low bond strength. Although air drying can help to remove the water within the adhesive and dentin, and facilitate adhesive polymerization, it can hardly remove all the water in cavities with relatively complex shapes [26]. Furthermore, over-thining of the adhesive layer by excessive drying is detrimental to successful bonding [28].

Compared with one-step self-etching adhesives, two-step self-etching adhesive systems contain higher amount of hydrophobic monomers. Therefore, two-step self-etching adhesives presents less phase-separation and can form a more uniform adhesive layer with less amount of retained water and solvent, resulting in higher bond strength. The functional monomer 10-MDP in SEB can interact with hydroxyapitite to form stable molecules like calcium phosphate and calcium carbonate, which can present as a resistant layer (approximately 4 nm), that can protect the bonded interface from aging-induced failure [2]. Interestingly, it was found in this study that the bond strength of SEB after four months of aging was similar to that of GB. Presumably, this is caused by the technique sensitivity of air drying. While gentle air drying may not be able to remove the solvents in the adhesive, excessive air drying may blow away the primer before it can sufficiently demineralize and prime the dentin surface. Either condition may contribute to an imperfect hybrid layer that is susceptible to collagen degradation and failure.

Although it was found by this study that both the types of adhesive systems and the period of aging have significant influence on bond strength, the reduction in micro-tensile bond strength over time are not very remarkable as compared to other studies. This maybe related to the relatively high standard deviation of the data for micro-tensile bond strength, as well as the short aging period.

In summary, therefore, negative correlation was definitely exist between type I collagen degradation and durability of dentin-adhesive interface. However, it can be stated that collagen degradation can be just regarded as one of the influencing factors. Other factors, such as the quality of dentin and hydrolysis of polymerized resin network, are also important influences for the stability of dentin-adhesive interface. Further studies will be performed to study other influencing factors and their combined effects on the durability of bonded resin restoratives.

## References

[1] Van Meerbeek B, De Munck J, Yoshida Y, Inoue S, Vargas M, et al (2003) Buonocore memorial lecture. Adhesion to enamel and dentin: current status and future challenges. Oper Dent 28: 215–235. 12760693

[2] Yoshida Y, Nagakane K, Fukuda R, Nakayama Y, Okazaki M, et al (2004) Comparative study on adhesive performance of functional monomers. J Dent Res 83: 454–458. 15153451 10.1177/154405910408300604

[3] Perdigão J (2004) Dentin bonding-variables related to the clinical situation and the substrate treatment. Dent Mater 26: e24–e37. 10.1016/j.dental.2009.11.149 20005565

[4] Reis A, Loguereio AD, Carvalho RM, Grande RH (2004) Durability of resin dentin interfaces: effects of surface moisture and adhesive solvent component. Dent Mater 20: 669–676. 15236942 10.1016/j.dental.2003.11.006

[5] Marshall GW, Marshall SJ, Kinney JH, Balooch M (1997) The dentin substrate structure and properties related to bonding. J Dent 25: 441–458. 9604576 10.1016/s0300-5712(96)00065-6

[6] Kinney JH, Balooch Marshall SJ, Marshall GW, Weihs TP. (1996) Atomic force microscope measurements of the hardness and elasticity of peritubular and intertubular human dentin. J Biomech Eng 118: 133–135. 8833085 10.1115/1.2795939

[7] Pashley DH (1991) The clinical correlation of dentin structure and function. J Prosthet Dent 66: 777–781. 1805028 10.1016/0022-3913(91)90414-r

[8] De Munck J, Mine A, Van den Steen PE, Van Landuyt KL, Poitevin A, et al (2010) Enzymatic degradation of adhesive–dentin interfaces produced by mild self-etch adhesives. Eur J Oral Sci 118: 494–501. 10.1111/j.1600-0722.2010.00758.x 20831584

[9] De Munck J, Vander Steen PE, Mine A, Van Landuyt KL, Poitevin A, et al Inhibition of enzymatic degradation of adhesive-dentin interfaces. J Dent Res 88: 1101–1106. 10.1177/0022034509346952 19861692

[10] Yang B, Adelung R., Ludwig K, Bossmann K, Pashley D H, et al (2005) Effect of structural change of collagen fibrils on the durability of dentin bonding. Biomaterials 26: 5021–5031. 15769538 10.1016/j.biomaterials.2005.01.024

[11] Zheng X, Pan H, Wang Z, Chen H (2011) Real-time enzymatic degradation of human dentin collagen fibrils exposed to exogenous collagenase: an AFM study in situ. Journal of Microscopy 241: 162–170. 10.1111/j.1365-2818.2010.03412.x 21118210

[12] Hashimoto M, Fujita S, Nagano F, Ohno H, Endo K (2010) Ten-years degradation of resin–dentin bonds. Eur J Oral Sci 118: 404–410. 10.1111/j.1600-0722.2010.00744.x 20662915

[13] Breschi L, Gobbi P, Mazzotti G, Falconi M, Ellis TH, et al (2002) High resolution SEM evaluation of dentin etched with maleic and citric acid. Dent Mater 18: 26–35. 11740962 10.1016/s0109-5641(01)00017-3

[14] Poitevin A, De Munck J, Van Landuyt K, Coutinho E, Peumans M, et al (2008) Critical analysis of the influence of different parameters on the microtensile bond strength of adhesives to dentin. J Adhesiv Dent 10: 7–16. 18389731

[15] Pashley DH, Tay FR, Yiu C, Hashimoto M, Breschi L, et al (2004) Collagen degradation by host-derived enzymes during aging. J Dent Res 83: 216–221. 14981122 10.1177/154405910408300306

[16] Klont B, ten Cate JM (1990) Release of organic matrix components from bovine incisor roots during in vitro lesion formation. J Dent Res 69: 896–900. 2324354 10.1177/00220345900690031301

[17] Bhattacharjee A, Bansal M. (2005) Collagen structure: the Madras triple helix and the current scenario. IUBMB Life 57: 161–172. 16036578 10.1080/15216540500090710

[18] Inoue S, Vargas MA, Abe Y, Yoshida Y, Lambrechts P, et al (2001) Micro-tensile bond strength of eleven contemporary adhesives to dentin. J Adhes Dent 3:237–45. 11803711

[19] Shono Y, Terashita M, Shimada J, Kozono Y, Carvalho RM, et al (1999) Durability of resin-dentin bonds. J Adhes Dent 1: 211–218. 11725669

[20] De Munck J, Van Meerbeek B, Yoshida Y, Inoue S, Vargas M, et al (2003) Four-year water degradation of total-etch adhesives bonded to dentin. J Dent Res 82: 136–140. 12562888 10.1177/154405910308200212

[21] Zhang S-C, Kern M (2009) The role of host-derived dentinal matrix metalloproteinases in reducing dentin bonding of resin adhesives. Int J Oral Sci 1: 163–176. 10.4248/IJOS.09044 20690420 PMC3470104

[22] Pashley DH, Tay FR, Breschi L, Tjäderhane L, Carvalho RM, et al (2011) State of the art etch-and-rinse adhesives, Dent Mater 27: 1–16 10.1016/j.dental.2010.10.016 21112620 PMC3857593

[23] Scott JE, Thomlinson AM (1998) The structure of interfibrillar proteoglycan bridges (‘shape modules’) in extracellular matrix of fibrous connective tissues and their stability in various chemical environments. J Anat 192: 391–405. 9688505 10.1046/j.1469-7580.1998.19230391.x PMC1467783

[24] Spencer P, Wang Y (2002) Adhesive phase separation at the dentin interface under wet bonding conditions. J Biomed Mater Res 62: 447–456. 12209931 10.1002/jbm.10364

[25] Shin TP, Yao X, Huenergardt R, Walker MP, Wang Y (2009) Morphological and chemical characterization of bonding hydrophobic adhesive to dentin using ethanol wet bonding technique. Dent Mater, 25: 1050–1057. 10.1016/j.dental.2009.03.006 19371945 PMC2748385

[26] Van Meerbeek B, Yoshihara K, Yoshida Y, Mine A, De Munck J, et al (2011) State of the art of self-etch adhesives. Dent Mater 27: 17–28. 10.1016/j.dental.2010.10.023 21109301

[27] Van Landuyt KL, DeMunck J, Snauwaert J, Coutinho E, Poitevin A, et al (2005) Monomer-solvent phase separation ine one-step self-etch adhesives. J Dent Res 84: 183–188. 15668338 10.1177/154405910508400214

[28] Spreafico D, Stefano S, Dario M, Dino R, Massimo G, et al (2006) The effect of the air-blowing step on the technique sensitivity of four different adhesive systems. J Dent 34: 237–244. 16202499 10.1016/j.jdent.2005.06.004

